# Diagnostic Performance of Cerebrospinal Fluid Neurofilament Light Chain and Soluble Amyloid-β Protein Precursor β in the Subcortical Small Vessel Type of Dementia

**DOI:** 10.3233/JAD-230680

**Published:** 2023-12-06

**Authors:** Elin Axelsson Andrén, Petronella Kettunen, Maria Bjerke, Sindre Rolstad, Henrik Zetterberg, Kaj Blennow, Anders Wallin, Johan Svensson

**Affiliations:** a Department of Internal Medicine and Clinical Nutrition, Institute of Medicine, Sahlgrenska Academy, University of Gothenburg, Gothenburg, Sweden; b Department of Psychiatry and Neurochemistry, Institute of Neuroscience and Physiology, Sahlgrenska Academy, University of Gothenburg, Mölndal, Sweden; c Department of Psychiatry, Cognition and Old Age Psychiatry, Sahlgrenska University Hospital, Region Västra Götaland, Mölndal, Sweden; d Laboratory of Clinical Neurochemistry, Department of Clinical Biology, Universitair Ziekenhuis Brussel, and Center for Neurosciences (C4N), Vrije Universiteit Brussel, Brussels, Belgium; e Department of Biomedical Sciences and Institute Born-Bunge, University of Antwerp, Antwerp, Belgium; f Department of Psychology, Faculty of Social Science, University of Gothenburg, Gothenburg, Sweden; g Clinical Neurochemistry Labratory, Sahlgrenska University Hospital, Mölndal, Sweden; h Department of Neurodegenerative Disease, Institute of Neurology, University College London, London, UK; i UK Dementia Research Institute at University College London, London, UK; j Hong Kong Center for Neurodegenerative Diseases, Hong Kong, China; k Wisconsin Alzheimer’s Disease Research Center, University of Wisconsin School of Medicine and Public Health, University of Wisconsin-Madison, Madison, WI, USA; l Department of Internal Medicine, Skaraborg Central Hospital, Region Västra Götaland, Skövde, Sweden

**Keywords:** Alzheimer’s disease, biomarkers, cerebrospinal fluid, neurofilament light chain, soluble amyloid-β protein precursor β, subcortical small vessel type of dementia

## Abstract

**Background:**

The subcortical small vessel type of dementia (SSVD) is a common subtype of vascular dementia, but there is a lack of disease-specific cerebrospinal fluid (CSF) biomarkers.

**Objective:**

We investigated whether CSF concentrations of neurofilament light chain (NFL), soluble amyloid-β protein precursor α (sAβPPα), sAβPPβ, and CSF/serum albumin ratio could separate SSVD from healthy controls, Alzheimer’s disease (AD), and mixed dementia (combined AD and SSVD).

**Methods:**

This was a mono-center study of patients with SSVD (*n* = 38), AD (*n* = 121), mixed dementia (*n* = 62), and controls (*n* = 96). The CSF biomarkers were measured using immunoassays, and their independent contribution to the separation between groups were evaluated using the Wald test. Then, the area under the receiver operating characteristics curve (AUROC) and 95% confidence intervals (CIs) were calculated.

**Results:**

Elevated neurofilament light chain (NFL) and decreased sAβPPβ independently separated SSVD from controls, and sAβPPβ also distinguished SSVD from AD and mixed dementia. The combination of NFL and sAβPPβ discriminated SSVD from controls with high accuracy (AUROC 0.903, 95% CI: 0.834–0.972). Additionally, sAβPPβ combined with the core AD biomarkers (amyloid-β_42_, total tau, and phosphorylated tau_181_) had a high ability to separate SSVD from AD (AUROC 0.886, 95% CI: 0.830–0.942) and mixed dementia (AUROC 0.903, 95% CI: 0.838–0.968).

**Conclusions:**

The high accuracy of NFL and sAβPPβ to separate SSVD from controls supports that SSVD is a specific diagnostic entity. Moreover, SSVD was distinguished from AD and mixed dementia using sAβPPβ in combination with the core AD biomarkers.

## INTRODUCTION

The subcortical small vessel type of dementia (SSVD), an often underdiagnosed subtype of vascular cognitive impairment, may comprise half of all patients with vascular dementia (VaD) [1, 2]. SSVD progresses gradually, and the clinical characteristics includes executive dysfunction, deficient self-control, mood disturbances, mild to moderate memory loss, and eventually functixonal disability. These features correlate with subcortical vascular changes like white matter hyperintensities (WMHs) on magnetic resonance imaging (MRI). Furthermore, vascular disease of the SSVD type might accelerate the progression of Alzheimer’s disease (AD) [3–5], and the state of combined AD and vascular pathologies could be denominated as mixed dementia [3, 4]. However, it may be difficult to separate SSVD from AD and normal aging based on cognitive profile and MRI characteristics [4, 6, 7]. At present, there are no CSF biomarker or combination of biomarkers with high diagnostic accuracy for SSVD. Therefore, the establishment of biomarkers alongside neuroimaging, neuropsychology, and clinical assessment could add to disease definitions at the SSVD-AD-spectrum and improve the diagnostics of SSVD. Also, biomarkers specific for SSVD could result in the inclusion of well-defined patient cohorts in clinical studies, potentially serve as surrogates for disease progression, and add further knowledge of the mechanisms underlying SSVD.

Neurofilament light chain (NFL) reflects changes in the brain white matter and subcortical axonal neurodegeneration [8, 9]. Consequently, NFL is not a disease-specific biomarker, and elevated NFL concentrations have been reported in several cognitive disorders engaging subcortical brain regions [10–12]. In AD, CSF NFL concentrations are typically moderately increased [10, 13, 14]. In the LeukoAraiosis and Disability Study (LADIS), higher CSF NFL concentration was associated with higher amount of WMHs, a proxy of SSVD [15, 16]. In a systematic review and meta-analysis, CSF NFL concentration was found to be increased in VaD, but subtypes of VaD were not analyzed [8]. In earlier studies using a less sensitive NFL assay with high detection limit, CSF NFL concentrations were increased in VaD populations comprising a varying degree of SSVD patients [12–14, 17, 18]. Also, in recent studies using a sensitive enzyme-linked immunosorbent assay (ELISA), CSF NFL concentrations were increased in well-defined SSVD populations [19, 20]. However, the diagnostic utility of CSF NFL, alone or in combination with other biomarkers, has not been evaluated in detail in SSVD.

Soluble amyloid-β protein precursor (sAβPP) α and sAβPPβ are, together with other AβPP metabolites such as amyloid-β_42_ (Aβ_42_), generated by the cleavage of the membrane protein AβPP. Except for Aβ_42_, most studies have found unchanged levels of AβPP metabolites in AD [21]. Recently, decreased CSF sAβPPβ concentration was found in SSVD patients, but the ability of sAβPPβ to separate SSVD from controls was moderate [22].

The underlying pathologies of SSVD include arteriolosclerosis, lipohyalinosis, fibroid necrosis, edema, and damage to the blood-brain barrier (BBB), resulting in chronic leakage of fluid and macromolecules in the brain white matter, and subsequent demyelination [3, 4, 23]. Thus, BBB dysfunction may be involved in SSVD development, and the best biomarker to date of BBB function is the CSF/serum albumin ratio [24]. In previous studies, the CSF/serum albumin ratio tended to be increased [20], or was significantly elevated [22], in SSVD patients.

In summary, although SSVD is associated with specific features in terms of cognitive profile and MRI characteristics, the lack of SSVD-specific CSF biomarkers can result in diagnostic challenges. Therefore, in this cross-sectional study performed at a single memory clinic, we included patients with SSVD, AD, and mixed dementia (combined AD and SSVD) as well as healthy controls. We investigated if the CSF biomarkers NFL, sAβPPα, sAβPPβ, and CSF/serum albumin ratio, alone or in combination, could separate SSVD patients from healthy controls. Furthermore, we also investigated if these biomarkers could distinguish SSVD from AD and mixed dementia.

## MATERIALS AND METHODS

### Participants and setting

The participants were recruited from the Gothenburg mild cognitive impairment (MCI) study, a prospective mono-center study conducted at the memory clinic at Sahlgrenska University Hospital, Mölndal, Sweden [6, 7]. Patients underwent baseline examinations including lumbar puncture to evaluate the degree and cause of cognitive impairment and were then followed every second year. Inclusion criteria were age 50–79 years, Mini-Mental State Examination (MMSE) score > 18, and self- or informant-reported cognitive decline≥6 months. Exclusion criteria were states that could affect cognitive decline such as severe somatic diseases (subdural hemorrhage, brain tumor, hypothyroid state, encephalitis, and unstable heart disease) as well as psychiatric disorders (major affective disorder, schizophrenia, substance abuse, and confusion). The exclusion criterion of unstable heart disease was defined as the presence of any ongoing symptoms of angina pectoris or myocardial infarction. Cognitively unimpaired elderly controls were recruited from senior citizen organizations (e.g., at information meetings on dementia), and a small proportion were relatives of patients. Exclusion criteria, as well as the study procedures, were similar to those of the patients [6, 7]. In the present study, we included all patients in the Gothenburg MCI study with a diagnosis of SSVD, AD, or mixed dementia as well as all controls that had an available CSF sample for analysis of the studied biomarkers.

The patients were classified using the Global Deterioration Scale (GDS), in which GDS 4 equals possible mild dementia and GDS 1 equals no subjective or objective cognitive decline [25]. The classification into GDS groups were based on medical history, checklists, and instruments for cognitive symptoms [6]: 1) Stepwise Comparative Status Analysis (STEP) variables 13–20 [26]; 2) I-FLEX, a short form of the Executive Interview (EXIT) [27]; 3) MMSE [28]; and 4) Clinical Dementia Rating (CDR) [29]. Guidelines for GDS 4 was: STEP > 1, I-FLEX > 3, CDR > 1.0, and MMSE≤25. However, a consensus decision among the specialized physicians was made to determine the appropriate GDS score.

In patients with GDS 4, the physician that determined the specific dementia diagnoses had access to clinical symptomatology and WMH amount (Fazekas scale [30]) on MRI but was blinded to neuropsychological test results and CSF biomarker data. However, in the present study, we also performed subanalyses in which we excluded SSVD patients and controls with CSF biomarker criteria for AD (Aβ_42_ < 530 ng/L, total (t)-tau > 350 ng/L and phosphorylated (p)-tau_181_ > 59 ng/L) [31]. We defined SSVD according to the Erkinjuntti criteria [32]. More specifically: a SSVD patient had to have MRI-detected cerebral WMHs (mild, moderate, or severe according to Fazekas classification [31]) and predominant frontal lobe symptoms. If WMHs were only mild, then SSVD was set only if parietotemporal lobe symptoms (such as apraxia, aphasia, and agnosia) were not marked. AD was diagnosed according to the NINCDS-ADRDA criteria [33]. Mixed dementia was diagnosed if AD patients also fulfilled the criteria of SSVD [6].

The classification used in the present study is in line with the results of the Vascular Impairment of Cognition Classification Consensus Study (VICCCS) in which SSVD, denominated subcortical ischemic vascular dementia, is one of the entities [34]. In the current analysis, we included patients with a baseline diagnosis of SSVD (*n* = 26) as well as patients with a baseline diagnosis of AD (*n* = 84) or mixed dementia (*n* = 42). In addition, to extend the study population, we also included baseline values from patients with MCI that had converted to SSVD (*n* = 12), AD (*n* = 37), or mixed dementia (*n* = 20) at the 2-year visit. Thus, 96 healthy controls and 221 patients (SSVD, *n* = 38; AD, *n* = 121; and mixed dementia, *n* = 62) were included. We excluded 37 patients with other forms of dementia (cortical stroke-related VaD, primary progressive aphasia, Lewy body dementia, frontotemporal dementia, or unspecified dementia).

### Ethical considerations

The study was approved by the regional ethical committee (diary number: L091-99 and T479-11) and the Swedish Ethical Review Authority (diary number: 2020-06733). The research was conducted according to the Declaration of Helsinki. Written informed consent was obtained from all participants.

### Cardiovascular risk factors

At the inclusion visit, a memory clinic physician recorded presence of hypertension, diabetes mellitus (henceforth: diabetes), obesity, and hyperlipidemia. Arterial hypertension was defined as antihypertensive treatment or blood pressure≥140/90 or≥130/80 for diabetics at repeated measurements [35]. Diabetes was defined, according to the 2006/2011 World Health Organization (WHO) diagnostic criteria [36], as fasting plasma glucose≥7.0 mmol/l (126 mg/dl), two-hour plasma glucose≥11.1 mmol/l (200 mg/dl) during oral glucose tolerance test, or treatment with oral antidiabetics and/or insulin. BMI≥30 kg/m^2^ was defined as obesity [37]. Hyperlipidemia was defined based on the European Society of Cardiology (ESC)/European Atherosclerosis Society (EAS) criteria [38], but modified according to local guidelines [6, 7]; we defined serum low density lipoprotein (LDL)-cholesterol≥3.0 mmol/l and/or serum triglycerides≥1.8 mmol/l as hyperlipidemia.

### Neuropsychological tests

In addition to the tests used for GDS classification, visual scanning and complex attention were evaluated using the Trail Making Test A (TMT-A) and B (TMT-B) [39]. Episodic memory was assessed using the delayed recall from the Rey Auditory Verbal Learning Test (RAVLT) [40].

### White matter hyperintensities

MRI was performed using a 1.5 T Siemens Symphony (Erlangen, Germany). WMH volumes were assessed using the FreeSurfer automated segmentation software (version 5.3.0; https://surfer.nmr.mgh.harvard.edu/) [6]. The individual WMH volumes were adjusted for intracranial volume (ICV) as described previously [41].

### Blood and CSF samples

After an overnight fast, blood samples were obtained between 8 a.m. and 10 a.m. and CSF samples between 8 a.m. and 12 a.m. to reduce diurnal fluctuations. Lumbar puncture was performed at the lumbar interspace between vertebrae L3 to L5, and CSF was collected in polypropylene tubes. The first tube of CSF was discarded to avoid blood contamination. Then, 20 mL of CSF was collected and centrifuged at 2,000×g for 10 min in room temperature, and then stored in cryo tubes at –80°C until analyses [6].

### Biochemical methods

The CSF analyses were performed at the Clinical Neurochemistry Laboratory, Sahlgrenska University Hospital, Mölndal, Sweden, by laboratory technicians who were blinded to clinical information. CSF NFL was measured using a sensitive sandwich ELISA (NF-light® ELISA kit, UmanDiagnostics AB, Umeå, Sweden). The lower limit of quantification was 31 ng/L. CSF sAβPPα and sAβPPβ were measured using the sAβPPα/sAβPPβ duplex assay (Meso Scale Diagnostics, Rockville, MD, USA). CSF AD biomarkers (Aβ_42_, t-tau, and p-tau_181_) were measured using INNOTEST® ELISA assays (Fujirebio, Ghent, Belgium). To ensure test quality, two internal control samples (aliquots of pooled CSF) were analyzed in each run. Intra- and inter-assay coefficients of variation were lower than 10%. Serum and CSF albumin concentrations were measured using immunonephelometry on a Beckman Immage immunochemistry system (Beckman Instruments, Beckman Coulter, Brea, CA). *APOE* genotyping in blood was performed in 299 of the 317 participants (Table 1) by mini-sequencing [6].

**Table 1:** Characteristics of patients and controls at baseline

Variable	SSVD (*n* = 38)	AD (*n* = 121)	Mixed dementia	Control	*p* between
			(*n* = 62)	(*n* = 96)	groups
Men/women (*n*, %)	25/13 (66/34)^a,b,c^	44/77 (36/64)	21/41 (34/66)	38/58 (40/60)	0.007
Age (y)	79 (66–75)^b,d^	66 (62–72)^a,f^	71 (67–75)^d^	64 (60–69)	<0.001
MMSE score	26 (25–28)^d^	26 (24–28)^d^	26 (23–27)^d^	30 (29–30)	<0.001
TMT-A (s)	59 (45–85)^d^	54 (44–73)^d^	57 (45–76)^d^	34 (27–41)	<0.001
TMT-B (s)	172 (120–243)^d^	141 (105–202)^d^	157 (118–300)^d^	78 (68–95)	<0.001
RAVLT delayed recall score	4 (1–7)^b,d^	2 (0–3)^d^	1 (0–4)^d^	9 (6–12)	<0.001
Education (y)	11 (8.5–13)	10 (8.5–14)	10.5 (7–13)	12 (10–14)	0.09
Smoking, yes/no (*n*, %)	6/32 (16/84)	14/107 (12/88)	9/53 (14/86)	12/82 (13/87)	0.89
White matter hyperintensities (cm^3^)	12.3 (5.9–27.7)^d,e^	3.4 (2.2–4.1)^a,f^	7.3 (4.5–12.3)^d^	1.9 (1.5–4.0)	<0.001
Number of *APOE4* alleles, 0/I/II (*n*, %)	4/19/14 (11/51/38)^c^	23/35/57 (20/30/50)^d^	15/16/29 (25/27/48)^d^	3/54/30 (3/62/35)	<0.001

Values are given as the median (25th–75th percentiles), or in numbers (n) and percentages (%) for categorical variables. Between-group differences were analyzed using the Kruskal-Wallis test followed by post hoc analyses using the Mann-Whitney U test. ^a^*p* < 0.05 versus control, ^b^*p* < 0.05 versus AD, ^c^*p* < 0.05 versus mixed dementia, ^d^*p* < 0.001 versus control, ^e^*p* < 0.001 versus AD, ^f^*p* < 0.001 versus mixed dementia. SSVD, subcortical small vessel disease; AD, Alzheimer’s disease; MMSE, Mini-Mental State Examination; TMT, Trail Making Test; RAVLT, Rey Auditory Verbal Learning Test.

### Statistical analyses

The statistical analyses were performed using SPSS version 28 (IBM Corp., Armonk, NY). The descriptive data are presented as the median and 25^th^–75^th^ percentiles. Between-group differences were assessed using the Kruskal-Wallis test followed by post hoc analyses using the Mann-Whitney U test. Correlations were evaluated using the Spearman rank order correlation test.∥The independent contribution of the biomarkers to the separation between two study groups were determined using backward stepwise binary logistic regression (Wald test). The relationships between sensitivity and specificity between study groups were analyzed using receiver operation characteristics (ROC) analysis. In these analyses, we calculated area under the receiver operating characteristics curve (AUROC) and 95% confidence intervals (CIs). The significance level was set to *p* < 0.05.

## RESULTS

### Clinical characteristics

Clinical characteristics are given in Table 1. Male sex was more frequent in the SSVD group than in the other study groups. Median age was higher in the patient groups than in the control group, and in addition, the SSVD and mixed dementia groups had higher age than the AD group. The neuropsychological test scores (MMSE, TMT-A, TMT-B, and RAVLT delayed recall) were impaired in all patient groups, but the SSVD group had less impaired RAVLT delayed recall score than the AD group. The educational level and current smoking did not differ across groups. All patient groups had higher WMH volumes than the control group, and the SSVD and mixed dementia groups in addition had higher WMH volume than the AD group. The AD and mixed dementia groups had higher prevalence of *APOE* ɛ4 carriership than the controls, and *APOE* ɛ4 carriership was also more common in mixed dementia than in SSVD.

Hypertension was more common in SSVD [*n* = 26 (68%)] than in AD [*n* = 21 (17%), *p* < 0.001], mixed dementia [*n* = 23 (37%), *p* = 0.01], and controls [*n* = 19 (20%), *p* < 0.001] (data not shown). The prevalence of diabetes was higher in the SSVD group [*n* = 5 (13%)] compared with that in AD [*n* = 4 (3%), *p* < 0.05] and controls [*n* = 2 (2%), *p* < 0.05], but there was no statistical difference compared with mixed dementia [*n* = 3 (5%), *p* = 0.14]. The prevalence of hypertension and diabetes was statistically similar in AD, mixed dementia, and controls. Obesity (BMI≥30 kg/m^2^) [SSVD, *n* = 7 (18%); AD, *n* = 7 (6%); mixed dementia; *n* = 4 (6%); controls, *n* = 7 (7%)] and hyperlipidemia [SSVD, *n* = 24 (63%); AD, *n* = 89 (74%); mixed dementia; *n* = 47 (76%); controls, *n* = 69 (72%)] were statistically similar in all study groups.

### CSF biomarkers

In terms of the core AD biomarkers (Table 2 and Supplementary Fig. 1), all dementia groups had lower CSF Aβ_42_ concentrations than the control group, and SSVD patients had higher Aβ_42_ concentration than patients with mixed dementia. The AD and mixed dementia groups had higher t-tau and p-tau_181_ concentrations than the SSVD and control groups.

**Fig. 1:**
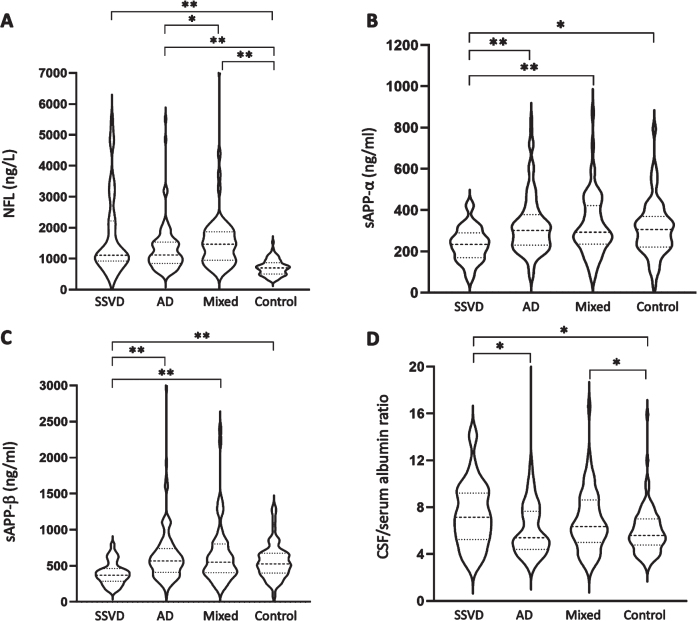
CSF biomarker concentrations are altered in SSVD. CSF median concentrations (25th–75th percentiles) are given for (A) NFL, (B) sAβPPα, (C) sAβPPβ, and (D) CSF/serum albumin ratio in patients with SSVD (*n* = 38), AD (*n* = 121), and mixed dementia (*n* = 62) as well as healthy controls (*n* = 96). The dashed lines represent the median and the scattered lines represent the percentiles. Between-group differences were assessed using the Kruskal-Wallis test followed by post hoc analyses using the Mann-Whitney U test. ^*^p < 0.05, ^**^p < 0.001. CSF, cerebrospinal fluid; SSVD, subcortical small vessel disease; AD, Alzheimer’s disease; Mixed, mixed dementia; NFL, neurofilament light chain; sAβPP, soluble amyloid-β protein precursor

All dementia groups displayed higher CSF NFL concentrations than the control group (Table 2 and Fig. 1A). Also, the mixed dementia group had higher NFL concentration than the AD group. CSF sAβPPα and sAβPPβ concentrations were lower in the SSVD group compared with the other study groups (Table 2 and Fig. 1B, C). The SSVD and mixed dementia groups had higher CSF/serum albumin ratio than the control group, and in addition, the SSVD group had higher CSF/serum albumin ratio than the AD group (Table 2 and Fig. 1D).

**Table 2:** Baseline levels of cerebrospinal fluid (CSF) biomarkers

Variable	SSVD (*n* = 38)	AD (*n* = 121)	Mixed dementia (*n* = 62)	Control (*n* = 96)	*p* between groups
Aβ_42_ (ng/L)	593 (425–700)^a,f^	386 (314–500)^d^	395 (318–486)^d^	658 (500–870)	<0.001
t-tau (ng/L)	319 (240–408)^e,f^	610 (420–840)^d^	700 (460–873)^d^	245 (170–359)	<0.001
p-tau_181_ (ng/L)	49 (37–61)^e,f^	82 (62–113)^d^	80 (61–103)^d^	47 (34–61)	<0.001
NFL (ng/L) ^#^	1109 (921–2217)^d^	1119 (848–1533)^c,d^	1464 (949–1889)^d^	701 (505–863)	<0.001
sAβPPα (ng/mL)	233 (170–290)^a,e,f^	301 (230–378)	293 (235–422)	306 (221–369)	<0.001
sAβPPβ (ng/mL)	371 (286–462)^d,e,f^	568 (410–739)	551 (405–803)	526 (400–676)	<0.001
CSF/serum albumin ratio	7.2 (5.3–9.2)^a,b^	5.4 (4.4–7.7)	6.4 (5.0–8.6)^a^	5.6 (4.8–7.0)	0.01

### Accuracy of CSF biomarkers to separate SSVD from controls

**Fig. 2:**
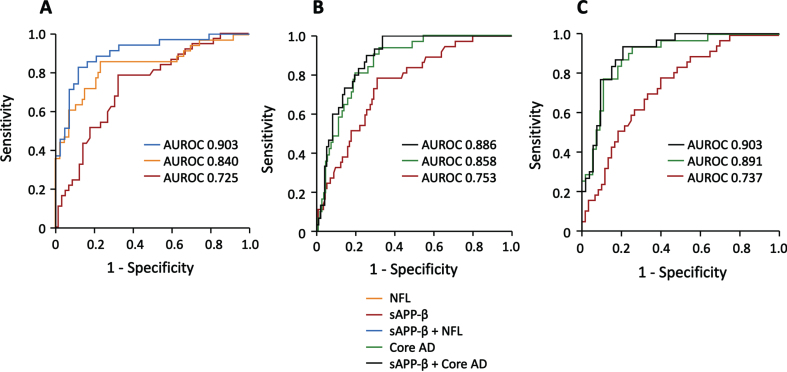
SSVD can be separated from AD, mixed dementia, and healthy controls using CSF biomarkers. The results of ROC statistics are presented. A) The separation of SSVD from healthy controls by NFL (AUROC 0.840, 95% CI: 0.754–0.926, *p* < 0.001), sAβPPβ (AUROC 0.725, 95% CI: 0.620–0.829, *p* < 0.001), and the combined use of NFL and sAβPPβ (AUROC 0.903, 95% CI: 0.834–0.972, *p* < 0.001). B) The separation of SSVD from AD by sAβPPβ (AUROC 0.753, 95% CI: 0.668–0.838, *p* < 0.001), the combined use of the core AD biomarkers Aβ_42_, t-tau and p-tau_181_ (AUROC 0.858, 95% CI: 0.794–0.923, *p* < 0.001), and the combined use of sAβPPβ and the core AD biomarkers (AUROC 0.886, 95% CI 0.830–0.942, *p* < 0.001). C) The separation of SSVD from mixed dementia by sAβPPβ (AUROC 0.737, 95% CI: 0.638–0.835, *p* < 0.001), the core AD biomarkers (AUROC 0.891, 95% CI: 0.823–0.960, *p* < 0.001), and the combined use of sAβPPβ and the core AD biomarkers (AUROC 0.903, 95% CI 0.838–0.968, *p* < 0.001). In the panels, orange represents NFL; red, sAβPPβ; blue, sAβPPβ combined with NFL; green, the core AD biomarkers; black, sAβPPβ combined with the core AD biomarkers. SSVD, subcortical small vessel disease; AD, Alzheimer’s disease; CSF, cerebrospinal fluid; ROC, receiver operating characteristics; AUROC, area under the receiver operating characteristics; NFL, neurofilament light chain; sAβPP, soluble amyloid-β protein precursor; Aβ_42_, amyloid-β_42_; t-tau, total tau; p-tau_181_, phosphorylated tau_181_.

Using ROC statistics, we found that NFL (AUROC 0.840, 95% CI: 0.754–0.926, *p* < 0.001) had a relatively high ability to distinguish SSVD from controls (Fig. 2A). In contrast, sAβPPβ (AUROC 0.725, 95% CI: 0.620–0.829, *p* < 0.001; Fig. 2A), sAβPPα (AUROC 0.690, 95% CI: 0.583–0.797, *p* < 0.01) and CSF/serum albumin ratio (AUROC 0.658, 95% CI: 0.546–0.771, *p* < 0.01) had lower diagnostic accuracy.

Then, backward stepwise binary logistic regression (Wald test) was used to identify whether NFL, sAβPPα, sAβPPβ, or CSF/serum albumin ratio could independently discriminate SSVD from controls. The Wald test identified NFL and sAβPPβ as independent contributors, and further ROC analyses showed that the combined use of NFL and sAβPPβ had high diagnostic accuracy (AUROC 0.903, 95% CI: 0.834–0.972, *p* < 0.001) (Fig. 2A). Furthermore, in the ROC analysis, the optimal cutoff points for NFL and sAβPPβ was 875 ng/L (sensitivity 86.1%, specificity 76.7%) and 466 ng/mL (sensitivity 78.4%, specificity 67.3%), respectively. In the analysis of NFL in combination with sAβPPβ, the sensitivity was 82.9% and the specificity was 88.6% at the optimal cutoff point.

In terms of the core AD biomarkers, Aβ_42_ had low ability to distinguish SSVD from controls (AUROC 0.623, 95% CI: 0.520–0.725, *p* < 0.01), whereas t-tau and p-tau_181_ had no significant discriminatory ability. The addition of Aβ_42_ alone (AUROC 0.896, 95% CI: 0.823–0.969, *p* < 0.001) or all three AD biomarkers (AUROC 0.915, 95% CI: 0.848–0.983, *p* < 0.001) only marginally affected the diagnostic accuracy of the combined NFL and sAβPPβ (not shown).

### Accuracy of CSF biomarkers to separate SSVD from AD and mixed dementia

In terms of separation of SSVD from AD, sAβPPβ showed moderate diagnostic accuracy (AUROC 0.753, 95% CI: 0.668–0.838, *p* < 0.001). sAβPPα (AUROC 0.711, 95% CI: 0.620–0.802, *p* < 0.001) and CSF/serum albumin ratio (AUROC 0.638, 95% CI: 0.534–0.742, *p* = 0.01) had some discriminatory ability, whereas the separation by NFL was not significant (data not shown). Finally, sAβPPβ was the only biomarker that independently separated SSVD from AD using the Wald test (Fig. 2B).

The SSVD group was distinguished from the mixed dementia group with moderate diagnostic accuracy by sAβPPβ (AUROC 0.737, 95% CI: 0.638–0.835, *p* < 0.001) and sAβPPα (AUROC 0.696, 95% CI: 0.592–0.799, *p* = 0.001). The classifications by NFL or CSF/serum albumin ratio were not significant. Using the Wald test, only sAβPPβ independently discriminated SSVD from mixed dementia (Fig. 2C).

Next, we evaluated whether the addition of sAβPPβ to the core AD biomarkers could improve the diagnostic accuracy. In terms of the separation of SSVD from AD, the diagnostic accuracy of the three AD biomarkers (AUROC 0.858, 95% CI: 0.794–0.923, *p* < 0.001) improved after the addition of sAβPPβ (AUROC 0.886, 95% CI: 0.830–0.942, *p* < 0.001) (Fig. 2B). Also, in the discrimination of SSVD from mixed dementia, the diagnostic accuracy of the core AD biomarkers (AUROC 0.891, 95% CI: 0.823–0.960, *p* < 0.001) was improved by the addition of sAβPPβ (AUROC 0.903, 95% CI 0.838–0.968, *p* < 0.001) (Fig. 2C).

### Accuracy of CSF biomarkers to separate AD from mixed dementia

NFL (AUROC 0.612, 95% CI: 0.518–0.706, *p* = 0.02), but not sAβPPα, sAβPPβ, or CSF/serum albumin ratio, distinguished AD from mixed dementia (not shown). However, using the Wald test, none of these biomarkers had an independent ability to separate AD from mixed dementia.

### Correlations

Correlation analyses were performed between NFL, sAβPPα, sAβPPβ, and CSF/serum albumin ratio in the entire population (*n* = 319) and in the SSVD group (*n* = 38). In the total study population, CSF/serum albumin ratio correlated positively with NFL (*r* = 0.20, *p* < 0.001) and negatively with sAβPPβ (*r* = –0.14, *p* = 0.02), and sAβPPα correlated positively with sAβPPβ (*r* = 0.70, *p* < 0.001). In the SSVD group, the only observed correlation was the positive one between sAβPPα and sAβPPβ (*r* = 0.55, *p* < 0.001).

### Sub-analyses of the diagnostic accuracy of the combined NFL and sAβPPβ

We performed sub-analyses to evaluate if the CSF AD biomarker pattern or age affected the ability of the combined NFL and sAβPPβ to separate SSVD from control. In a first analysis, we excluded five SSVD patients and five controls with CSF biomarker criteria for AD (Aβ_42_ <530 ng/L, t-tau > 350 ng/L and p-tau_181_ > 59 ng/L). In the remaining CSF AD biomarker negative participants, the combination of NFL and sAβPPβ still distinguished SSVD from controls with high diagnostic accuracy (AUROC 0.885, 95% CI: 0.804–0.944, *p* < 0.001).

In a second sub-analysis, as CSF NFL concentration increases with age, we split the SSVD and control groups with a cutoff point of 70 years. In the participants aged below 70 years, the median (25^th^ –75^th^ percentiles) age in SSVD (*n* = 19) and the controls (*n* = 78) did not differ between groups [*p* = 0.10; SSVD: 66 (60–68) years and controls: 63 (57–66) years]. Likewise, in the SSVD patients (*n* = 19) and the relatively few controls (*n* = 18) with age above 70 years, there was no between-group difference in age [*p* = 0.08; SSVD: 75 (71–77) years and controls: 73 (70–75) years]. Also, in these age-matched analyses, NFL in combination with sAβPPβ separated SSVD from controls with high accuracy in the younger participants (AUROC 0.890, 95% CI: 0.804–0.975, *p* < 0.001) as well as in the older participants (AUROC 0.944, 95% CI: 0.839–1.000, *p* = 0.02).

## DISCUSSION

In this mono-center study of memory clinic patients, we investigated whether CSF concentrations of NFL, sAβPPα, sAβPPβ, and CSF/serum albumin ratio could separate SSVD from healthy controls, AD, and mixed dementia. The SSVD group had decreased CSF concentrations of sAβPPα and sAβPPβ, and increased CSF concentrations of NFL and CSF/serum albumin ratio compared with the control group. In addition, sAβPPα and sAβPPβ concentrations were decreased in the SSVD group compared with the AD and mixed dementia groups. Using the Wald test, both NFL and sAβPPβ had independent abilities to separate SSVD from controls, and in the ROC analyses, the combined use of NFL and sAβPPβ distinguished SSVD from controls with high diagnostic accuracy. At the optimal cutoff point, the combined NFL and sAβPPβ had a sensitivity of 82.9% and a specificity of 88.6% for the discrimination of SSVD from controls. Finally, sAβPPβ in combination with the core AD biomarkers (Aβ_42_, t-tau and p-tau_181_) had a high capacity to discriminate SSVD from AD and mixed dementia.

A major finding of the present study is that the combined use of NFL and sAβPPβ can discriminate SSVD from cognitively healthy controls with high diagnostic accuracy. The high ability of the combined NFL and sAβPPβ to separate SSVD from controls remained also after exclusion of participants that had CSF biomarker criteria for AD as well as in the sub-analyses of age-matched SSVD patients and controls. Previously, increased CSF NFL concentrations have been found in VaD and mixed dementia [8, 10, 13], which is in accordance with our finding of higher CSF NFL concentration in mixed dementia compared with AD. Furthermore, in the LADIS cohort [42], CSF NFL concentration was positively associated with the degree of WMHs (a proxy of SSVD) at baseline, whereas the association with WMH progression was weaker [15, 16, 43]. CSF NFL concentrations have also been increased in recent studies of well-defined SSVD patients using a sensitive ELISA assay [19, 20], but the diagnostic utility has not been evaluated. Our results therefore extend the previous knowledge by demonstrating that NFL alone has a relatively high, and the combination of NFL and sAβPPβ have a high, ability to discriminate SSVD from cognitively healthy controls.

In the present study, CSF concentrations of sAβPPα and sAβPPβ were lower in the SSVD group than in the other study groups, but only sAβPPβ contributed independently to the separation between groups using the Wald test. Previously, lower sAβPPβ concentrations have been associated with increased amount of WMHs on MRI [16, 43]. Also, sAβPPβ concentration was decreased in post-stroke patients compared with other patients having subjective or mild objective cognitive symptoms [44]. In two recent studies in SSVD, CSF concentrations of soluble AβPPs were decreased [19, 22], but the ability of sAβPPβ to separate SSVD from controls was moderate [22]. In the present study, we show that sAβPPβ used in combination with NFL has a high ability to distinguish SSVD from healthy controls and that the addition of sAβPPβ to the core AD biomarkers improves the separation of SSVD from AD and mixed dementia.

In the brain, neurofilaments are expressed in mature neurons where they form fibrillary networks, which provide structural stability and resistance against mechanical stress to axons [45–47]. Furthermore, in large myelinated axons, neurofilaments are involved in radial growth, dendritic branching, axonal transport, and regulation of the position of cellular organelles [45–47]. Less is known of the role of sAβPPβ in the brain. However, in neuronal cultures, sAβPPβ decreased cell adhesion and increased axon elongation [48]. In addition, sAβPPβ can interact with death receptor 6 (DR6) in the brain, thereby regulating neuronal cell death and axonal pruning [49]. Thus, NFL and sAβPPβ might reflect different processes in the brain and could therefore also be markers of different pathological events in SSVD development. Speculatively, diverging nature of the biomarkers might underlie that the combined use of them, but neither of the biomarkers alone, separated SSVD from healthy controls with high diagnostic accuracy.

In the current study, CSF/serum albumin ratio was higher in the SSVD and mixed dementia groups compared with the control group. In addition, the SSVD group had a higher CSF/serum albumin ratio than the AD group. As the CSF/serum albumin ratio is a marker of BBB function [24], our findings are in line with the notion that BBB dysfunction is involved in SSVD development by causing chronic leakage of fluid and macromolecules that damage the brain white matter [4]. However, we did not find that the CSF/serum albumin ratio was able to independently separate SSVD from the other study groups. In a previous study, CSF/serum albumin ratio was higher in cognitively impaired patients with WMHs on MRI compared with cognitively healthy patients and patients with AD characteristics [50]. In SSVD cohorts, the CSF/serum albumin ratio tended to be increased [20] or was significantly elevated [22], but the diagnostic ability to separate SSVD from controls was modest [22]. Overall, our results suggest that although the CSF/serum albumin ratio is increased in SSVD, it adds small value to the distinction of SSVD from AD, mixed dementia, or controls.

In our SSVD group, CSF Aβ_42_ concentration was lower than in the controls, whereas it was higher than in mixed dementia. Similarly, in a previous study, CSF Aβ_42_ concentration was lower in MCI patients who later converted to SSVD than in controls, but higher than in MCI patients developing AD or mixed dementia [18]. Most [51–53], but not all [20] earlier studies have shown moderately reduced CSF Aβ_42_ concentrations in VaD populations comprising a varying degree of SSVD patients compared with controls. Furthermore, in cerebral autosomal dominant arteriopathy with subcortical infarcts and leukoencephalopathy (CADASIL) [54], a group in which confounding AD is unlikely, CSF Aβ_42_ concentration was lower than in controls [55]. So, compared with controls, there are several indications that subcortical small vessel disease is associated with moderately reduced CSF Aβ_42_ concentration. Yet, the addition of Aβ_42_ to the combination of NFL and sAβPPβ did not improve the separation of SSVD from controls in our study.

Patients with mixed dementia (combined AD and SSVD) had higher CSF NFL concentration and higher CSF/serum albumin ratio than the controls and in addition, NFL was higher in mixed dementia than in AD. However, in contrast to the findings in SSVD patients, CSF concentrations of sAβPPα and sAβPPβ were unchanged in mixed dementia compared with both AD patients and controls, and patients with mixed dementia had similar concentrations of the core CSF AD biomarkers (Aβ_42_, t-tau, and p-tau_181_) as AD patients. Therefore, it appears as if the CSF biomarker profile in mixed dementia is more in resemblance with that of AD than with that of SSVD. In support of this assumption, none of the studied CSF biomarkers independently separated mixed dementia from AD. This is in turn in accordance with the previous observations that the presence of AD neuropathology is of more importance for the clinical phenotype than the existence of concomitant SSVD [56, 57]. Regardless, there is a need for further studies to find biomarkers that can distinguish between mixed dementia and AD.

In the present study, the neuropsychological test results confirmed differences between the SSVD patients and the controls with worsened scores of TMT-A and TMT-B and moderately impaired RAVLT delayed recall score in the SSVD patients. However, although there was a small numerical difference in TMT-B score, the TMT-A and TMT-B scores were not significantly different in the SSVD group compared with the AD and mixed dementia groups. The latter finding may be surprising as executive dysfunction and reduced mental speed are considered as typical for SSVD [34]. The reason for this is unknown, but there is a possibility that the TMT-A and TMT-B tests do not reflect the full spectrum of executive dysfunction in SSVD. However, as expected, the RAVLT delayed recall score reflecting episodic memory was more impaired in the AD group compared with the SSVD group.

The results of the present study, if confirmed in future studies, suggest that NFL and sAβPPβ can be used in the clinical setting alongside other clinical and neuroimaging assessments to separate SSVD from healthy individuals. Particularly, measurements of NFL and sAβPPβ could be of value when the diagnosis of SSVD is not straightforward due to modest or moderate amount of MRI-estimated brain WMHs and/or a cognitive profile that is not typical. Furthermore, the core AD biomarkers (Aβ_42_, t-tau, and p-tau_181_) in combination with sAβPPβ had a high ability to separate SSVD from AD and mixed dementia. At present there is no curative treatment for SSVD. Nevertheless, a correct diagnosis is important to ensure that a patient receive as optimal care as possible. Furthermore, when recruiting patients to clinical studies of novel medical treatments for AD and other conditions, it is vital to include patients with correct diagnoses to have adequate results of the studies and avoid unnecessary adverse effects. In such situations, the benefits of improved diagnostics of SSVD are most likely greater than the risk of side effects of lumbar puncture such as transient headache and local pain. Better diagnostic procedures could also result in increased knowledge of the mechanisms underlying SSVD, which could in turn generate disease-specific biomarkers with even higher diagnostic accuracy than that seen in present study as well as new prevention strategies and treatments.

Major strengths of the present study are the mono-center design and the extensive characterization of the included patients and controls. We excluded patients with stroke related cortical VaD, thereby having one study group consisting only of SSVD patients. Furthermore, we extended the study population by including baseline values from patients with a baseline dementia diagnosis as well as baseline values from patients that had converted to SSVD, AD, or mixed dementia at the 2-year follow up. Nonetheless, the limited number of patients might have reduced the statistical power. We did not correct for multiple comparisons. However, our main analysis was to determine whether NFL, sAβPPα, sAβPPβ, and CSF/serum albumin ratio could separate SSVD from healthy controls, and the ability of NFL and sAβPPβ to separate these two groups was highly significant and would have remained also after correction for multiple comparisons. Also, we cannot evaluate cause-and-effect relationships due to the cross-sectional design. Consequently, longitudinal studies are needed to determine whether the CSF concentrations of the biomarkers are altered during the progression of the SSVD disease.

In summary, as there are no established CSF biomarkers, the diagnosis of SSVD is not straightforward. The results of our mono-center study suggest that CSF concentrations of NFL and sAβPPβ used in combination can distinguish SSVD from cognitively healthy controls with high diagnostic accuracy, which supports the notion that SSVD is a specific diagnostic entity. Furthermore, the core AD biomarkers had a high capacity to separate SSVD from AD and mixed dementia, especially when used in combination with sAβPPβ. If these results are confirmed in future studies, there is a possibility that biomarker criteria can be applied in the diagnostic procedures of SSVD as a complement to clinical and neuroimaging methods in memory clinic patients. Future studies are also needed to evaluate if measurements of biomarkers in blood can separate SSVD from AD, mixed dementia, and healthy controls.

## Supplementary Material

Supplementary Material

## Data Availability

The data supporting the findings of this study are available on request from the corresponding author. The data are not publicly available due to privacy or ethical restrictions.
