# Supplementary material for: Diagnostic Performance of Cerebrospinal Fluid Neurofilament Light Chain and Soluble Amyloid-β Protein Precursor β in the Subcortical Small Vessel Type of Dementia
Source: J Alzheimers Dis. 2023 Dec 6;96(4):1515–28. doi: 10.3233/JAD-230680 (PMC10741327; doi:10.3233/JAD-230680)
Supplement: Supplementary Material [file jad-96-jad230680-s001.pdf]

Supplementary Material

Diagnostic Performance of Cerebrospinal Fluid Neurofilament Light Chain and Soluble Amyloid-β Protein Precursor β in the Subcortical Small Vessel Type of Dementia

Supplementary Figure 1. CSF concentrations of the core AD biomarkers are different in SSVD compared with AD, mixed dementia, and healthy controls. CSF concentrations are given for (A) $A\beta_{42}$, (B) t-tau, and (C) p-tau_{181} in patients with SSVD (n = 38), AD (n = 121), and mixed dementia (n = 62) as well as healthy controls (n = 96). The short horizontal lines represent the median values, and the longer dashed lines represent the cutoff values for each biomarker. Between-group differences were assessed using the Kruskal-Wallis test followed by post hoc analyses using the Mann-Whitney U test.

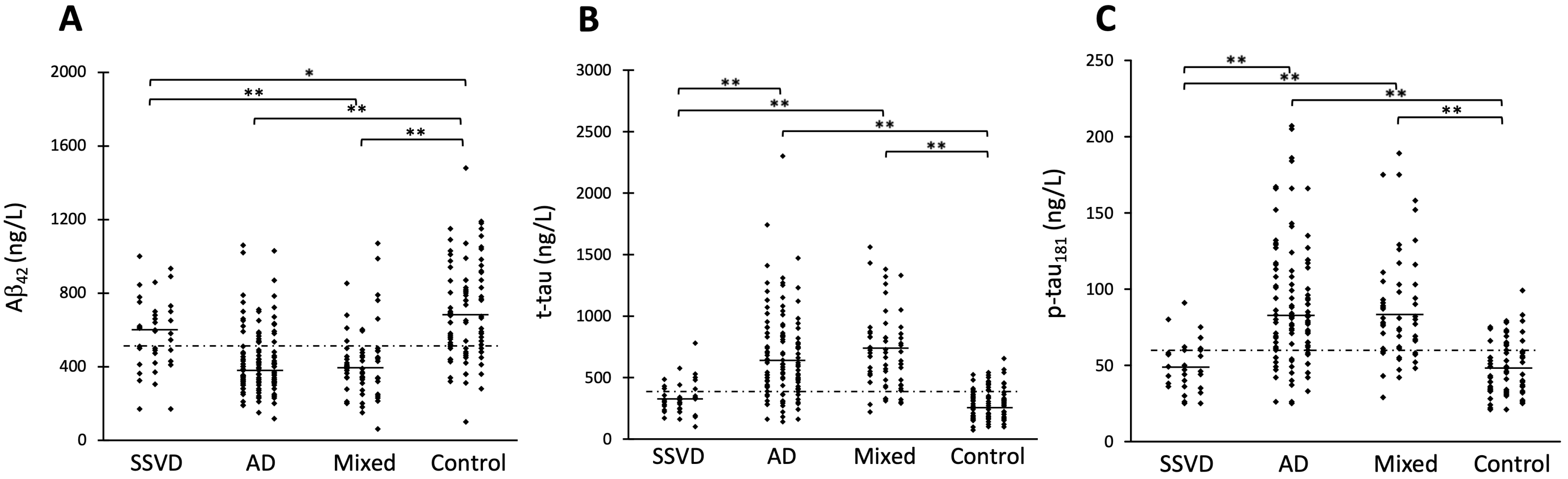

* $p < 0.05$, ** $p < 0.001$

CSF, cerebrospinal fluid; SSVD, subcortical small vessel disease; AD, Alzheimer's disease; Mixed, mixed dementia; $A\beta_{42}$, amyloid-β_{42}; t-tau, total tau; p-tau_{181}, phosphorylated tau_{181}
